# VARAdb: a comprehensive variation annotation database for human

**DOI:** 10.1093/nar/gkaa922

**Published:** 2020-10-23

**Authors:** Qi Pan, Yue-Juan Liu, Xue-Feng Bai, Xiao-Le Han, Yong Jiang, Bo Ai, Shan-Shan Shi, Fan Wang, Ming-Cong Xu, Yue-Zhu Wang, Jun Zhao, Jia-Xin Chen, Jian Zhang, Xue-Cang Li, Jiang Zhu, Guo-Rui Zhang, Qiu-Yu Wang, Chun-Quan Li

**Affiliations:** School of Medical Informatics, Daqing Campus, Harbin Medical University. Daqing 163319, China; School of Medical Informatics, Daqing Campus, Harbin Medical University. Daqing 163319, China; School of Medical Informatics, Daqing Campus, Harbin Medical University. Daqing 163319, China; School of Medical Informatics, Daqing Campus, Harbin Medical University. Daqing 163319, China; School of Medical Informatics, Daqing Campus, Harbin Medical University. Daqing 163319, China; School of Medical Informatics, Daqing Campus, Harbin Medical University. Daqing 163319, China; School of Medical Informatics, Daqing Campus, Harbin Medical University. Daqing 163319, China; School of Medical Informatics, Daqing Campus, Harbin Medical University. Daqing 163319, China; School of Medical Informatics, Daqing Campus, Harbin Medical University. Daqing 163319, China; School of Medical Informatics, Daqing Campus, Harbin Medical University. Daqing 163319, China; School of Medical Informatics, Daqing Campus, Harbin Medical University. Daqing 163319, China; School of Medical Informatics, Daqing Campus, Harbin Medical University. Daqing 163319, China; School of Medical Informatics, Daqing Campus, Harbin Medical University. Daqing 163319, China; School of Medical Informatics, Daqing Campus, Harbin Medical University. Daqing 163319, China; School of Medical Informatics, Daqing Campus, Harbin Medical University. Daqing 163319, China; School of Medical Informatics, Daqing Campus, Harbin Medical University. Daqing 163319, China; School of Medical Informatics, Daqing Campus, Harbin Medical University. Daqing 163319, China; School of Medical Informatics, Daqing Campus, Harbin Medical University. Daqing 163319, China

## Abstract

With the study of human diseases and biological processes increasing, a large number of non-coding variants have been identified and facilitated. The rapid accumulation of genetic and epigenomic information has resulted in an urgent need to collect and process data to explore the regulation of non-coding variants. Here, we developed a comprehensive variation annotation database for human (VARAdb, http://www.licpathway.net/VARAdb/), which specifically considers non-coding variants. VARAdb provides annotation information for 577,283,813 variations and novel variants, prioritizes variations based on scores using nine annotation categories, and supports pathway downstream analysis. Importantly, VARAdb integrates a large amount of genetic and epigenomic data into five annotation sections, which include ‘Variation information’, ‘Regulatory information’, ‘Related genes’, ‘Chromatin accessibility’ and ‘Chromatin interaction’. The detailed annotation information consists of motif changes, risk SNPs, LD SNPs, eQTLs, clinical variant-drug-gene pairs, sequence conservation, somatic mutations, enhancers, super enhancers, promoters, transcription factors, chromatin states, histone modifications, chromatin accessibility regions and chromatin interactions. This database is a user-friendly interface to query, browse and visualize variations and related annotation information. VARAdb is a useful resource for selecting potential functional variations and interpreting their effects on human diseases and biological processes.

## INTRODUCTION

Recent advances in high-throughput technologies, such as DNA sequencing and genome-wide association studies (GWAS), have generated a flood of data associating variants with complex human diseases and phenotypes (1). These variants often lie far from known genes, presumably in transcriptional regulatory regions, such as distal enhancers, transcription factor (TF) binding sites, and accessible chromatin regions (2). Several studies have shown that disease-associated variants were significantly enriched in regulatory elements that are dispersed widely across the genome. Many variants such as rs1198588, rs4442975 and rs117480515 have been found to affect TF binding sites within regulatory regions and regulate gene expression (3–5). Furthermore, variants nearby or located in enhancers were found to disrupt enhancer–promoter loop resulting in disease and gene expression dysregulation (6–9). For instance, rs4442975, a strong candidate for causality, flanks a transcriptional enhancer that physically interacts with the promoter of *IGFBP5*. Researchers have demonstrated that rs4442975 is associated with *FOXA1* binding and the low expression of *IGFBP5* increased breast cancer susceptibility. In colorectal cancer, risk SNP rs6983267 was found to increase *TCF7L2* binding and enhancer activity to regulate *c-MYC* expression (10,11). Therefore, annotation and analysis of non-coding variants can help to explain GWAS results and understand the genetic structure of diseases. Importantly, integrating variations with genetic and epigenomic information, and long-range interactions will help to select causal regulatory variants and understand underlying regulatory mechanisms in biological processes.

In recent years, several databases and web tools have been developed to annotate non-coding variants based on genetic and epigenomic information (12–15). HaploReg (12) and rSNPBase (15) are both useful databases for annotating cataloged variants. Different from HaploReg and rSNPBase, RegulomeDB can interrogate the regulatory information of novel variants by using a scoring system (13). These non-coding variant databases and web tools have provided effective platforms and available data for variant exploration. However, a flood of genomic datasets, such as enhancers, super enhancers, TFs, accessible chromatin regions and chromatin interactions, is accumulating rapidly, which promotes an urgent need to integrate and process the data for variants comprehensively and effectively. In particular, TFs from ChIP-seq data and motif changes of variants should be further provided. In addition, providing a strategy and regulatory network for variants based on genomic data will help researcher to prioritize potential functional variants in complex diseases. Fortunately, ENCODE (16), the Roadmap Epigenomics project (17), and UCSC (18), as well as other comprehensive data sources, have uncovered the landscape of massive regulatory elements in the genome. In addition, chromosome conformation capture (3C)-based technologies, Hi-C (19–21), ChIA-PET (22,23) and 5C (24), have provided increased datasets of chromatin interactions and revealed patterns on how regulatory elements regulate the expression of their target genes (20,25,26). Together, to integrate, prioritize and analyze variation-associated information, building a human variation annotation database is necessary. VARAdb will help to explore the regulatory mechanisms of variations and discover casual variations, which may accelerate the development of variation research.

To this end, we developed a comprehensive VARiation Annotation database for human (VARAdb, http://www.licpathway.net/VARAdb/), which is focused on providing a large number of variations and annotating their potential roles with various regulatory information, in particular, non-coding variants are considered. The current version of VARAdb cataloged a total of 577 283 813 variations and provided five annotation sections: ‘Variation information’, ‘Regulatory information’, ‘Related genes’, ‘Chromatin accessibility’ and ‘Chromatin interaction’. The genetic and epigenomic data involved motif changes, risk SNPs, linkage disequilibrium (LD) SNPs, eQTLs, clinical variant-drug-gene pairs, sequence conservation, somatic mutations, enhancers, super enhancers, promoters, TFs, chromatin states, histone modifications, ATAC accessible regions and chromatin interactions. Moreover, the database collected two types of variation-related genes: (i) variation related genes based on whether the variation is located in an enhancer or not (if it is, the variation may associate with enhancer target genes predicted by Lasso method) and (ii) variation-related genes based on distance including overlapping genes, proximal genes and the closest gene. In addition, to perform the in-depth analysis of variations, VARAdb was designed to prioritize variations based on score, annotate novel variants and provide pathway downstream analysis. VARAdb is a user-friendly database to query, browse and visualize information associated with variations. We believe that VARAdb could become a useful and effective platform for exploring potential functions and regulation of variations in human diseases and biological processes.

## MATERIALS AND METHODS

### Annotation of variation

**Figure 1.**
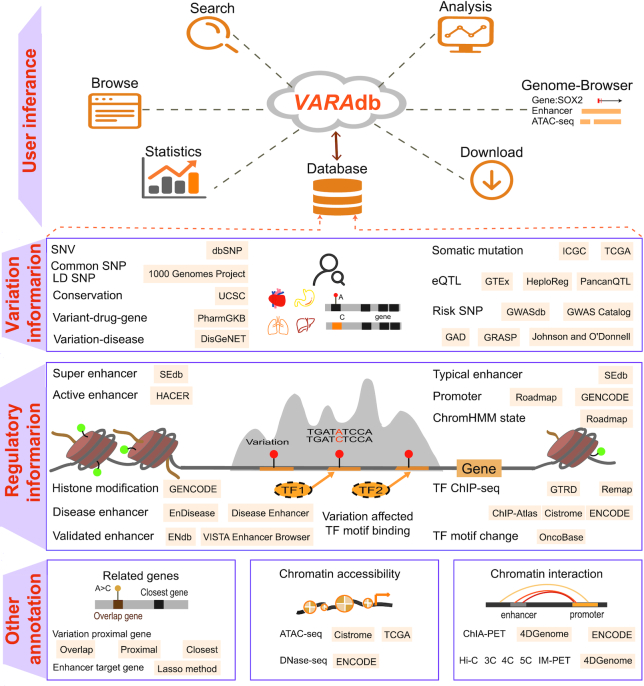
Database content and construction. VARAdb supports multiple functions including storage, search, analysis, browse, genome-browser, statistics and download. Massive genetic and epigenomic information as well as chromatin interactions are collected and integrated. VARAdb provides five annotation sections including ‘Variation information’, ‘Regulatory information’, ‘Related genes’, ‘Chromatin accessibility’ and ‘Chromatin interaction’, which involve motif changes, risk SNPs, LD SNPs, eQTLs, clinical variant-drug–gene pairs, sequence conservation, somatic mutations, enhancers, super enhancers, promoters, TFs, chromatin states, histone modifications, ATAC accessible regions and chromatin interactions. Annotation information is collected from multiple sources including eQTLs, somatic mutations, enhancers, promoters, TFs, variation proximal genes, GWAS data, ATAC data and ChIA-PET data.

We collected and integrated massive data of genetic and epigenomic information as well as chromatin interactions from various data sources, and divided annotation information into five sections including ‘Variation information’, ‘Regulatory information’, ‘Related genes’, ‘Chromatin accessibility’ and ‘Chromatin interaction’ (Figure 1). We describe these sections in detail below.

#### Variation information

To curate as many variations as possible, we collected variations from many resources including 577 098 938 human SNVs from the dbSNP release 151 (27), 79 482 384 common SNPs from 1000 Genomes Project (28), 1,515,001 risk SNPs from the GWAS Catalog (29), GWASdb v2.0 (30), GAD (31), Johnson and O’Donnell (32), and GRASP v2.0 (33), and 3 998 301 eQTLs from GTEx v7 (34), PancanQTL (35) and HaploReg v4.1 (12). In total, we collected 577 283 813 variations (10 386 595 coding region variations and 566 895 064 non-coding variations) and provided annotation information for each variation including eQTLs, LD SNPs, risk SNPs, sequence conservation, motif changes, somatic mutations, clinical variant-drug-gene pairs and variant–disease pairs.

##### eQTLs

The correlations between genotype and tissue-specific gene expression levels can interpret the effects of variants on genes using eQTL data in tissues or cancers. Human eQTL data were downloaded from GTEx v7 (34), PancanQTL (35) and HaploReg v4.1 (12). We downloaded and processed significant SNP–gene pairs in 48 human tissues from GTEx v7. For each eQTL pair, we annotated eQTL with ‘rsID’ according to ‘chr_position_ref_alt_b37’ and provided not only ‘Gene_ID’ but also ‘Gene_name’. Finally, we obtained 16 489 663 eQTL pairs (with false discovery rate, FDR ≤ 0.05, including 3 052 986 SNPs and 18 126 genes). From PancanQTL, we first obtained a large number of cis-eQTL pairs from 33 cancer types, then added three valuable statistical values to 5 596 894 cis-eQTL pairs (with FDR ≤ 0.05, 1 370 558 SNPs and 17 353 genes). At the same time, we collected 4 613 715 eQTL pairs (914 358 SNPs and 20 331 genes) in different tissues from HaploReg v4.1.

##### LD SNPs

LD SNPs may share similar regulatory information associated with a phenotype. We used VCFTools (v0.1.13) (36) and PLINK (v1.9) (37) to calculate LD SNPs for common SNPs accompanying the 1000 Genomes Project phase 3 (28). Using an LD threshold of *r*^2^ = 0.8 and a 200 kb window between variants, we obtained LD SNPs of five super-populations (AFR, African; AMR, Ad Mixed American; EAS, East Asian; EUR, European; SAS, South Asian).

##### Risk SNPs

GWAS have provided a large amount of data associating genetic variants with common phenotypes. We collected risk SNPs from five sources, including the NHGRI GWAS Catalog (29), GWASdb v2.0 (30), GAD (31), Johnson and O'Donnell (32), and GRASP v2.0 (33). Then, we filtered risk SNPs of which ‘Variant ID’ which are not ‘rsID’ and transformed locations of risk SNPs from the GRCh38 assembly version into the hg19 version in the GWAS Catalog. Finally, we obtained 1 515 001 risk SNPs associated with diseases, traits and phenotypes.

##### Sequence conservation

We obtained phastCons scores from multiple alignments of 100 vertebrate genomes in the UCSC browser and used the bigwigAverageOverBed tool to measure the conservation of each variation (38).

##### Motif changes

Predicting of the effects of variations on the binding of TFs is important to transcriptional regulation. The OncoBase (39) database uses motifbreakR (40) to measure the effects of somatic mutations on TF binding motifs that consists of 2 817 position weight matrices (PWMs) of TFs from four resources including ENCODE (16), FactorBook (41), HOCOMOCO (42) and Homer (43). To score and predict the effects of variation in our database, we added the motifbreak results from OncoBase to VARAdb.

##### Somatic mutations

Somatic mutations play vital roles in tumor development with specification. We added somatic mutations from OncoBase and divided them into two sources: 388 861 somatic mutations in 36 cancer types from TCGA (44) and 7 999 912 somatic mutations in 57 cancer types from ICGC (45).

##### Clinical variant-drug-gene pairs

Variant-drug pairs play an important role in medical treatments of various human diseases. From PharmGKB (46), we downloaded 3 652 human variant-drug pairs, which include 2 351 variants, 921 genes and 710 chemicals. Some results have been validated with various levels of evidence in PharmGKB.

##### Variant-disease associations

DisGeNET contains the largest publicly available collections of genes and variants associated to human disease, which integrates data from expert curated repositories, GWAS catalogues, animal models and the scientific Iiterature. We obtained 210 498 variant-disease associations between 117 337 variants and 10 358 diseases, traits and phenotypes from DisGeNET v6.0 (47).

#### Regulatory information

We used BEDTools (v2.25.0) (48) to annotate the variations with regulatory information. This annotation information involved four categories: super enhancers; enhancers; promoters, TFs identified by ChIP-seq, chromatin states and histone modifications. The details are described below.

##### Super enhancers

Super enhancers are large clusters of enhancers with a higher degree of enrichment for TFs, higher levels of transcription and stronger cell-type specificity (49). We obtained super enhancers involving 542 H3K27ac ChIP-seq samples from a SEdb database that was developed by our group (50). First, we collected H3K27ac ChIP-seq and their corresponding input control sequencing data from ENCODE (16), Roadmap (17), NCBI GEO/SRA (51), and Genomics of Gene Regulation Project (GGR) (16). Second, we used H3K27ac ChIP-seq raw fastq files as input to identify super enhancers controlling normalization and consistency across various data resources. Importantly, we learned and used the streamlined pipeline of Bowtie-MACS-ROSE, which was developed by Loven *et al.* (52). In detail, we ran Bowtie (53) to align all raw sequencing reads for each H3K27ac ChIP-seq file, and MACS (54) to call all peaks for the consistency of calling peaks. Then, we used the ROSE programs to identify super enhancers by stitching peaks (52).

##### Enhancers

To provide a comprehensive enhancer resource for variation annotation, the current release of VARAdb collected four major types of enhancers. They consist of 1 535 disease enhancers from DiseaseEnhancer (55) and EnDisease 2.0 (56), 1 416 experimentally validated enhancers from VISTA Enhancer Browser (57) and ENdb (58), 877 955 active enhancers from HACER GRO-seq/PRO-seq enhancers (59) and the FANTOM5 project (60), and 6 629 274 typical enhancers from SEdb that included 542 human H3K27ac samples from >240 tissues and cell types (50).

##### TFs identified by ChIP-seq

To validate the effects of variations on TF binding, we focused on TFs that were identified by ChIP-seq, which has been reported to be effective for validating the relationships between TFs and regulatory regions. From the TRlnc database that was also developed by our group, we obtained TFs from 7 734 ChIP-seq samples across 467 sample types from 44 tissues (61). We collected a total of 952 TFs from ENCODE (16), Remap (62), Cistrome (63), ChIP-Atlas (64) and GTRD (65). In VARAdb, we also considered the classifications of TFs including superclass, class, family and subfamily from TFClass database (66).

##### Chromatin states

Chromatin states, including enhancers, promoters, insulators and heterochromatin, were based on various histone modifications to analyze regulatory elements. From multiple chromatin marks, Roadmap used ChromHMM v1.10, a multivariate Hidden Markov method, to calculate chromatin states across 127 epigenomes. We added the ChromHMM core 15 states of five chromatin marks (H3K4me3, H3K4me1, H3K36me3, H3K27me3 and H3K9me3) to VARAdb. The core 15 states (67) include: 1_TssA (Active transcription start sites, TSS); 2_TssAFlnk (Flanking Active TSS); 3_TxFlnk (Transcr. at gene 5′ and 3′); 4_Tx (Strong transcription); 5_TxWk (Weak transcription); 6_EnhG (Genic enhancers); 7_Enh (Enhancers); 8_ZNF/Rpts (ZNF genes & repeats); 9_Het (Heterochromatin); 10_TssBiv (Bivalent/Poised TSS); 11_BivFlnk (Flanking Bivalent TSS/Enh); 12_EnhBiv (Bivalent Enhancer); 13_ReprPC (Repressed PolyComb); 14_ReprPCWk (Weak Repressed); 15_Quies (Quiescent/Low).

##### Promoters

We considered two types of promoters. One type was defined as 2 kb upstream and the other type was 1 kb downstream of TSSs. We determined the first type of promoters based on the basic gene annotation file of release 33 from GENCODE (68). The other was determined based on the promoter relevant states from ChromHMM core 15 states including 1_TssA (Active TSS) and 2_TssAFlnk (Flanking Active TSS).

##### Histone modifications

From ENCODE (16) and Roadmap (17), we obtained histone modifications (H3K36me3, H3K4me1, H3K4me3, H3K79me2, H4K20me1 and H3K9ac), which involved 686 ChIP-seq samples from 201 sample types and 50 tissue types. The biosample types of these include ‘Cell Line’, ‘Tissue’, ‘Primary Cell’ and ‘*In vitro* differentiated cells’.

#### Related genes

To enable the identification of related genes, eQTL genes from available resources and risk SNP-associated genes from GWAS were first considered because of their importance to human complex diseases and common phenotypes. We also considered another two strategies to obtain variation-related genes. One was the ROSE (52) method for predicting related genes based on distance, including overlapping genes, proximal genes, and the closest gene. The other was the Lasso algorithm for predicting enhancer target genes. We downloaded the relevant results based on the Lasso algorithm from a study by Cao *et al.* (69). When variations were located in the enhancers, the target genes of the enhancers were considered to be related to those variations.

#### Chromatin accessibility

Accessible chromatin regions can be identified using ATAC-seq and DNase-seq and have enriched multiple regulatory elements and variations. Regulatory elements located in the accessible chromatin regions were reported to associate with the regulation of the distal gene resulting in heterogeneity. We cataloged the ATAC-seq accessible regions of 99 samples from Cistrome (63) and 23 cancer types from TCGA (44), as well as DNase-seq accessible regions of 243 samples from ENCODE (16).

#### Chromatin interaction

Regulatory elements, such as enhancers and super enhancers, are anchored to the promoter regions of genes via chromatin looping to affect gene transcription. In VARAdb, we collected chromatin interaction data from three databases. We downloaded human chromatin interaction data of five types of experiments (Hi-C, ChIA-PET, IM-PET, 3C, 4C and 5C) from 4DGenome (70), ChIA-PET narrow peaks of five cell types (MCF-7, HeLa-S3, K562, NB4 and HCT116) from ENCODE (16), and chromatin interactions identified by the EpiTensor algorithm from OncoBase (39).

### Scoring strategy

In VARAdb, each variation was scored based on its annotated records on nine annotation categories: risk SNP, eQTL, motif change, conservation, enhancer/super enhancer, promoter, TF binding, ATAC accessible region and Hi-C. For a variation, we first organized its annotated records on different categories. Then we calculated the score of the variation, which is defined as follows:

(1) $$\begin{equation*}S{\rm{cor}}e(i) = \sum\limits_{k = 1}^9 {categor{y_k}(i)} \end{equation*}$$

where *Score(i)* ranges from 0 to 9, which is the number of categories the variation *i* is associated with. If variation *i* is associated with annotation category *k*, then the *category_k_(i)* is 1. Otherwise, the *category_k_(i)* is 0. The scoring strategy can help users to prioritize and filter the variations that are studied in their work.

## DATABASE USE AND ACCESS

### A search interface for retrieving variation

**Figure 2.**
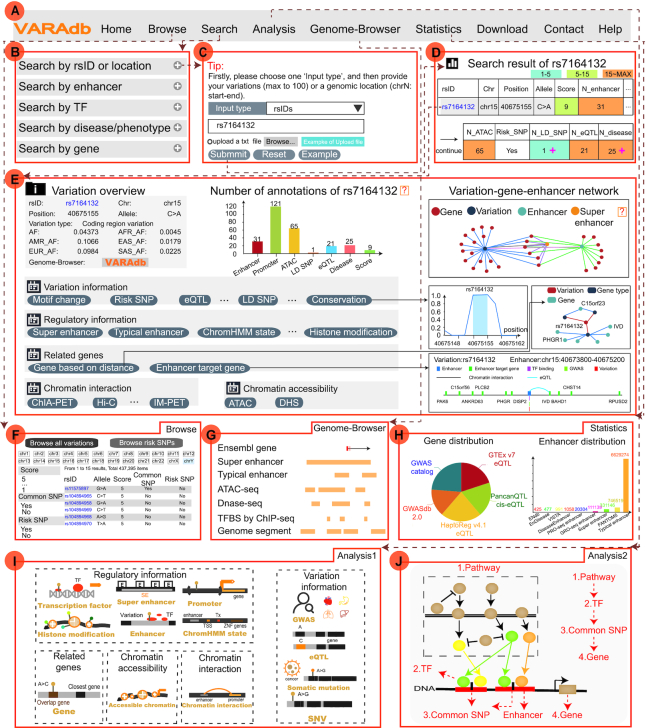
The main functions and usage of VARAdb. (**A**) The navigation bar of VARAdb. (**B**) Five query methods: ‘Search by rsID or location’, ‘Search by enhancer’, ‘Search by TF’, ‘Search by disease/trait/phenotype’, and ‘Search by gene’. (**C**) A detailed search, which is accomplished by inputting the ‘rsID’ or genomic location of variation. (**D**) The summary table that displays statistics about annotation information. (**E**) The detailed information of variation, which includes an overview of the variation, numbers of annotations of the variation, variation-gene-enhancer network, and five annotation results. (**F**) Browse page of VARAdb. (**G**) Genome-Browser of VARAdb. (**H**) Data statistics of VARAdb. (**I**) Novel variant annotation analysis. (**J**) Pathway downstream analysis.

VARAdb enables users to search, browse, analyze, visualize, and download variations of interest (Figure 2A). On the ‘search’ page, VARAdb provides five query methods for searching variation information. These include ‘Search by rsID or location’ (input rsIDs or a location of interest), ‘Search by enhancer’ (input enhancer location and select an enhancer type), ‘Search by TF’ (input a TF), ‘Search by disease/trait/phenotype’ (input disease/trait/phenotype name) and ‘Search by gene’ (input a gene) (Figure 2B). On the ‘Search by rsID or location’ results page, users can obtain a summary table of search results, which exhibits the statistics involving the detailed annotation of variation and description of each column, including rsID, chr, position, allele, score, enhancer, promoter, ATAC, common SNP, risk SNP, LD SNP, eQTL, disease/trait and variation type (Figure 2C, D; Supplementary Material 1). Users can click the ‘download’ button to download search results (Figure 2D) and click on ‘rsID’ the of summary table to view the details about each variation (Figure 2E). On the details page, VARAdb displays the variation overview, statistics charts, variation-gene-enhancer network and five annotation sections, these include: (i) regulatory information; (ii) variation information; (iii) related genes; (iv) chromatin accessibility and (v) chromatin interaction. In VARAdb, the different annotation sections have various annotating modules in VARAdb. For example, in the ‘Related genes’ section, users can select two modules to see genes associated with the variation (Figure 2E). Importantly, users can download the data table of interest on the details page by clicking on the ‘download’ button.

### User-friendly browsing

To allow users to browse all variations more conveniently and quickly, we have designed two modules ‘Browse all variations’ and ‘Browse risk SNPs’. The ‘Browse’ page is an interactive and alphanumerically sort table, from which users can use different conditions to filter variations and risk SNPs. When browsing all variations, users select at least one chromosome to browse variations and use ‘Score’, ‘Common SNP’, ‘Risk SNP’ and ‘Variation type’. In addition, risk SNPs show close relationships with different diseases, traits or phenotypes. When browsing the risk SNPs, users can use ‘Source’, ‘Score’, ‘Disease/trait/phenotype’ to search and filter risk SNPs risk SNPs with different scores in VARAdb (Figure 2F). For each risk SNP, the score ranges from 6 to 9 and was calculated based on annotation categories as previously described above. The ‘Show entries’ drop-down menu can change the number of records per page. In addition, users can view the details of risk SNPs by clicking on ‘rsID’.

### Data visualization

VARAdb exhibits different charts about data distribution and annotation statistics on the web interface. The relationships between variations and proximal genes, as well as between enhancers and proximal genes, are shown using a D3 network visualization plugin (Figure 2E). In addition, users can view regulatory information of variations in the genome by using the ‘Genome-Browser’ page. Through selecting useful tracks, users can further obtain detailed regulatory information, including common SNPs, conservation score, enhancers, nearby genes, conserved TF binding sites, TFBS by ChIP-seq, genome segments, ATAC accessible chromatin regions and DNase I hypersensitive sites (Figure 2G).

### Statistics and download

On the ‘Statistics’ page, users can see can see digital and graphical statistical displays in the VARAdb (Figure 2H). The ‘Download’ page exhibits ‘Variation information’, ‘Regulatory information’, ‘Related genes’, ‘Chromatin accessibility’ and ‘Chromatin interaction’ data for users to download. Moreover, the detailed descriptions of files are also displayed.

### Online analysis tools

#### Novel variant annotation

**Figure 3.**
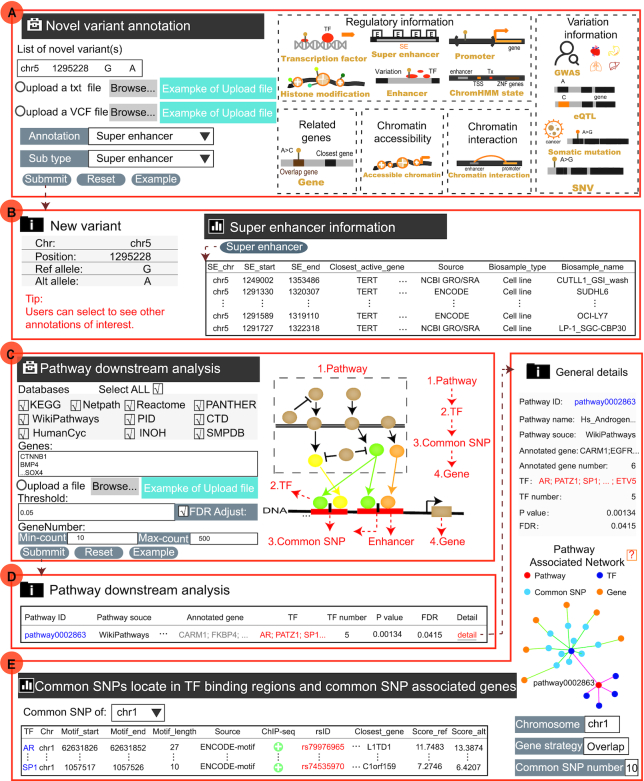
The two online analysis tools of VARAdb. (**A**) Novel variant annotation of VARAdb. There are five annotation sections: ‘Regulatory information’, ‘Variation information’, ‘Related genes’, ‘Chromatin accessibility’, and ‘Chromatin interaction’. (**B**) After submitting, VARAdb displays the detailed annotation results of the novel variation (chr5:1295228:G>A), which is a somatic mutation of the *TERT* promoter. (**C**) Pathway downstream analysis of VARAdb. (**D**) The output table displays basic information of enriched pathways, where *P* values are calculated by the hypergeometric test and FDR is used to correct for multiple testing. (**E**) After clicking on the detail button of the output table of (**D**) The results page provides general details of the enriched pathway, pathway-associated network, and information about common SNPs affecting TF binding and its proximal genes. The pathway-associated network is formed by pathway, TFs, common SNPs and common SNP-associated genes.

Recent advances in low-cost, high-throughput DNA sequencing have expanded genetic content and resulted in dramatically increased numbers of variants in the human genome. Most of the newly identified variants are non-coding variants, which are not collected and annotated in time. Moreover, biologists need to obtain various genetic and epigenomic information of the novel variants they want to explore. To strengthen the practical application of novel variants, we have designed ‘Novel variant annotation’ to provide five annotation sections, where the information includes accessible chromatin, chromHMM state, histone modification, TF, chromatin interaction, super-enhancer, promoter, gene, eQTL, GWAS, somatic mutation and SNV (Figure 2I; Figure 3A, B). User can not only manually input many novel variants (one variant with chrN position ref_allele alt_allele per line) but also upload a set of variants in a text file or a VCF file. For example, we have provided annotation information of a *TERT* promoter mutation (chr5:1295228:G>A) which is currently the best established example of recurrent cis-regulatory mutation found across various cancers (71). Users can first click ‘New variant annotation’ in ‘Analysis’, then input the genomic location (chr5:1295228) and the allele of the mutation (G>A), and select one annotation type (the default: Super enhancer) to start. On the results page, VARAdb not only displays the basic information about the mutation enriched in many super enhancers. In addition, users can directly select to see other annotation results by clicking on the nodes of the annotation image. VARAdb can help users investigate the potential functions of the novel variant according to annotation results with a friendly web interface.

#### Pathway downstream analysis

Regulatory elements are often enriched in many non-coding variants and play key roles in cancers. Especially, enhancers/super enhancers were reported to concentrate in multiple signaling pathways at key genes (72). The alterations of signaling pathways cover terminal DNA-bound TFs (73). In VARAdb, users can input genes of interest and select at least one pathway database (ten pathway databases including KEGG, Reactome, NetPath, WikiPathways, PANTHER, PID, HumanCyc, CTD, SMPDB, and INOH) to perform pathway downstream analysis based on the complex regulatory networks (74–84). Users can not only manually input genes (one gene symbol per line) but also upload a gene set in a text file. In addtion, VARAdb provided an apparent introduction to the function at the bottom of the database. When analyzing, VARAdb will provide different indications explaining why there are no results. After submitting, VARAdb will identify significantly enriched pathways, downstream TFs, common SNPs located in TF binding regions, and common SNP-associated genes (Figure 2J, Figure 3C). The enriched pathways are calculated using a hypergeometric test with the following formula:

(2) $$\begin{equation*}P = 1 - \sum\limits_{x = 0}^{m - 1} {\frac{{\left( {_x^k} \right)\left( {_{s - x}^{n - k}} \right)}}{{\left( {_s^n} \right)}}} \end{equation*}$$

where the *P* value is the enrichment significance of the pathway, *n* genes are in the entire genome, and *s* genes of interest are provided, of which *m* genes are involved in the pathway containing *k* genes. In addition, the FDR is also calculated to correct for multiple testing. VARAdb will show the output table containing summary information of enriched pathways including Pathway ID, Pathway name, Pathway source, Annotated gene, Annotated gene number, Total gene number, TF and TF number, *P* value, and FDR. Furthermore, the detailed description of the regulatory networks can be obtained by clicking the ‘Detail’ button (Figure 3D, E). Users may understand the roles of SNPs in the biological process based on affected TF motifs.

### Case study

To illustrate the usage of VARAdb, we searched the database with a well-studied variation rs2279590 (chr8:27456253:T>C, score = 6) by inputting the ‘rsID’ or its genomic location (Supplementary Figure S1A). The variation was associated with age-related disorders such as Alzheimer's disease (AD) and type 2 diabetes (85). We will validate the key predictions of the variation using VARAdb by collecting experimental data from studies. Notably, after clicking ‘rsID’ in the output table that first displayed the summary annotation information (Supplementary Figure S1B), the details page exhibited an overview of rs2279590, variation–gene–enhancer network, and five annotation sections (variation information, regulatory information, related genes, chromatin accessibility and chromatin interaction) (Supplementary Figure S1C). From ‘Variation information’ and ‘Related genes’, as a Risk SNP, rs2279590 was annotated as a genetic risk factor of AD and reported to regulate gene expression of *CLU*, which was consistent with studies of GWAS information (86,87). We found that *CLU* was the related gene of rs2279590 because *CLU* was not only the closest gene of rs2279590 but also an eQTL gene (Supplementary Figure S1C, left panel), which was validated by the experimental results of Padhy *et al.* (85). From ‘Regulatory information’, we found that rs2279590 was located in an enhancer of AD. Moreover, rs2279590 was enriched in super enhancers where *CLU* is the closest active gene. In addition, rs2279590 was associated with many histone marks such as H3K4me1 and H3K27ac as well as accessible chromatin regions (Supplementary Figure S1C, middle panel), which are in line with some studies (85). More importantly, the relationship between *CLU* and rs2279590-associated super enhancers was further validated by chromatin loops according to Hi-C and ChIA-PET data in the ‘Chromatin intersection’ (Supplementary Figure S1C, right panel).

The relationship between variation and TF is crucial to transcriptional regulation and worth detecting. From motif change data of ‘Variation information’, rs2279590 was predicted to strongly affect the binding of three TFs including *NFATC1*, *RFX1* and *SREBF1*. These TFs were regulated by other functional features such as receptors associated with AD (88–90) (Supplementary Figure S1C, left panel). For instance, N-methyl-D-aspartate receptors open channel blocker memantine is used to treat AD, which correlates with a reduction in T-cell receptor (TCR)-induced Ca(2+) mobilization and nuclear localization of *NFATC1* (89). From the TF ChIP-seq data of ‘Regulatory information’, VARAdb displayed rs2279590-associated TF binding information based on ChIP-seq data, which is an effective high-throughput experiment strategy for validating TF binding results (Supplementary Figure S1C, middle panel). We found that 14 out of 26 rs2279590-related TFs were reported to be associated with AD, these were *CEBPA*, *E2F6*, *EP300*, *ERG*, *ETS1*, *FOS*, *GATA2*, *IKZF1*, *NR4A1*, *RUNX1*, *SPI1*, *SPIB*, *TCF7L1* and *WT1* (91–104). For example, lower expression of TF *SPI1* reduces AD risk by regulating myeloid gene expression and cell function (96). We also found that TF *MAX* was associated with AD but not reported by in existing studies. *MAX* was enriched in AD-related Gene Ontology terms such as DNA binding and protein binding based on relevant results from ENSEMBL (105). In addition, these 14 TFs can be used to identify rs2279590-related pathways. After performing the ‘Pathway downstream analysis’, the output table showed that the ‘ERK cascade’, ‘Glucocorticoid receptor regulatory network’, and ‘Regulation of Androgen receptor activity’ were significantly enriched. Above all, these results showed the usefulness and value of VARAdb in exploring the potential function and regulation of variation.

Risk SNP rs6983267 (chr8:128413305:G>T, score = 6) was used as another example to access VARAdb. The variation is associated with colorectal cancer pathogenesis. After searching rs6983267, users can obtain various annotation results in the details page (Supplementary Figure S2A, B). We found that rs6983267 was predicted to affect the binding of TF *JUND*, which was further validated in HCT116 (sample type: cell line, tissue: colon) by TF ChIP-seq data from ‘Regulatory information’ (Supplementary Figure S2C, left panel). In colorectal cancer, rs6983267 was associated with *MYC*. Indeed, VARAdb identified *MYC* as a variation-related gene and enhancer-related target gene, which was consistent with previous studies (10,11). Notably, Wright *et al.* has presented evidence that the risk region harboring rs6983267 physically interacts with the *MYC* oncogene in colorectal cancer and rs6983267 binds to *TCF7L2* (10). In addition, an in vivo study by Sur *et al.* has reported that deletion of the enhancer region, including rs6983267, results in resistance to intestinal tumors (106). Interestingly, we found that rs6983267 was not only located in some super enhancers and typical enhancers identified by H3K27ac ChIP-seq data in colon relevant tissue and cell type, but also in many chromatin regions with the H3K4me1 mark (Supplementary Figure S2C, middle panel), as well as accessible regions in cholangiocarcinoma from ATAC-seq data of ‘Chromatin accessibility’ (Supplementary Figure S2C, right panel). A study by Wright *et al.* validated the predictions involving rs6983267, where this variation had enhancer-related histone marks such as H3K4me1 and was located in enhancers that form a 335-kb chromatin loop to interact with the *c-MYC* promoter (10). In addition, we also found that rs6983267 was associated with *PCAT1* which was not reported in existing studies. In VARAdb, *PCAT1* is an overlapping gene of rs6983267, which was reported to correlate with *MYC* (107). Moreover, GWAS data and other studies (10,11) showed that *MYC* was a rs6983267-associated gene. Therefore, these results suggest that VARAdb could explore the potential function and regulation of genes that are related to the variation.

## SYSTEM DESIGN AND IMPLEMENTATION

The current version of VARAdb was developed using MySQL 5.7.27 (http://www.mysql.com) and runs on a Linux-based Apache Web server (http://www.apache.org). We used PHP 5.6.40 (http://www.php.net) for sever-side scripting, Bootstrap v3.37 (https://v3.bootcss.com) and JQuerry v2.1.1 (http://jquery.com) for interactive interface building, Echarts (http://echarts.baidu.com) for visualization and JBrowse (http://jbrowse.org) for the genome browser. For the best display, we recommend using a comprehensive web server that supports HTML5 standard, for example, Firefox, Google Chrome, and Safari.

The research community can access information freely in the VARAdb database without registration or logging in. The URL for VARAdb is http://www.licpathway.net/VARAdb/.

## DISCUSSION

Expanding the functional datasets collected and integrating them across more effective resources will improve the functional predictions of non-coding variants in human complex diseases and phenotypes (108). The genetic and epigenomic information, as well as chromatin interactions, are important for interpreting the roles of non-coding variants enriched in various regulatory elements (109). Compared with the existing databases (12,13,15,109), VARAdb focuses on the comprehensive annotation of a large number of variations and massive regulatory annotation information. VARAdb not only provides annotation for the cataloged variations but also the novel ones. The current version of VARAdb cataloged a total of 577 283 813 variations which are ∼three times more than similar databases and provided five annotation sections including ‘Variation information’, ‘Regulatory information’, ‘Related genes’, ‘Chromatin accessibility’, and ‘Chromatin interaction’, with significantly more information than similar databases (Table 1).

**Table 1.** Comparison of VARAdb with other databases that provide annotations for variations

Function type	Data type/specific function	VARAdb	RegulomeDB	HaploReg v4.1	rSNPBase 3.0
**Annotation**	**Variation information**				
	Variations	577 283 813	22 164 519	52 054 804	117 452 549
	eQTL pairs	26 700 272^a^	142 945	2 415 272	4 201 218
	Risk SNP results	2 853 083^b^		✓	39 689
	Variant-drug-gene pairs	3 652			
	Motif change associated PWMs	2 817	1 158		
	Somatic mutations	✓			✓
	LD SNPs	✓		✓	✓
	Sequence conservation	✓	✓	✓	
	**Regulatory information**				
	Typical enhancers	6 629 274			
	Super enhancers	331 146			
	Active enhancers	877 955^c^			
	Disease enhancers	1 535^d^			
	Validated enhancers	1 416^e^	✓		
	ChromHMM core 15 states	✓		✓	
	Promoters	✓		✓	✓
	Histone modification conditions or cell lines	686	✓		
	TFs ChIP-seq samples	7 734		✓	
	**Related genes**				
	FANTOM5 enhancer-gene pairs	746 512^f^			
	Predicted enhancer-target gene pairs	5 134 313			
	Variation proximal genes^g^	✓			
	**Chromatin accessibility**				
	ATAC regions	4 232 806^h^			
	DHSs	✓	✓	✓	
	**Chromatin interaction**				
	Hi-C data	1 114 278			✓
	ChIA-PET data	682 526			✓
	IM-PET data	1 844 553			
	3C and 4C and 5C data	6 461			✓
**Analysis functions**	**Novel variant annotation analysis**	✓			
	**Pathway downstream analysis**	✓			
**Genome browser**	**DNA elements tracks for visualization**	✓			
**Score strategy**	**Contains one score strategy at least**	✓	✓		
**Browse**	**Browse** **a** **large** **number** **of variations**	✓			

^a^eQTL pairs were collected from three resources: GTEx v7, HaploReg v4.1 and PancanQTL.

^b^Risk SNP results were obtained from five resources: GWAS Catalog, GWASdb v2, GAD, Johnson and O’Donnell and GRASP.

^c^Active enhancers were obtained from HACER GRO-seq/PRO-seq data and FANTOM enhancers.

^d^Disease enhancers were collected from the DiseaseEnhancer and EnDisease databases.

^e^Validated enhancers were downloaded from VISTA ENHANCER BROWSER and ENdb.

^f^FANTOM5 enhancers proximal genes were predicted by the ROSE method and divided into three types: the closest, proximal and overlapping.

^g^Variation proximal genes were also predicted by the ROSE method and divided into three types: the closest gene, proximal genes and overlapping genes.

^h^Accessible regions predicted by ATAC-seq were obtained from Cistrome and TCGA.

VARAdb has rich annotations, scoring strategy, regulatory element information and useful analysis tools. Furthermore, VARAdb provides a user-friendly interface to browse, search, analyze, and visualize information about variations. Table 1, which compares VARAdb with other databases for information and functions, shows the advantages of VARAdb. We provide: (i) the annotation of 577 283 813 variations; (ii) the scores for 577 283 813 variations (range from 0 to 9) based on the annotated records of nine annotation categories: risk SNP, eQTL, motif change, conservation, enhancer/super enhancer, promoter, TF binding, ATAC accessible region and Hi-C; (iii) five annotation sections of variation, each of which contains detailed annotation information; (iv) online analysis tools including ‘Novel variant annotation’ and ‘Pathway downstream analysis’; (v) user-friendly visualization of genomic information of variants linking to the UCSC genome browser by adding multiple tracks; (vi) five search paths to access variation; (vii) user-friendly browsing; (viii) detail pages containing different charts for relationships between variations, risk SNPs, enhancers, genes and TFs.

VARAdb supports annotating the cataloged and novel variations in many categories. In the process of developing the database, we considered this matter. Because there are many data sources, we designed multiple sections and data modules for effectively managing updates in a modular way. This will effectively ensure the controllability of data and improve the efficiency of updating the database. Specifically, we designed five annotation sections: ‘Variation information’, ‘Regulatory information’, ‘Related genes’, ‘Chromatin accessibility’, and ‘Chromatin interaction’. For each section, we divided many sources of the same or similar characters into a group, which is an independent module. In other words, each module contains several data sources shown by a drop-down menu in the database. Because the stored information is based on the module of five sections, we can control the quality of data and ensure that VARAdb is user-friendly and effective. We developed a workflow for updating the database (Supplementary Figure S3). When updating each data source, the quality of data will be controlled according to the modular process. If a series of requirements are met, the data source will be updated and the score of variation may be also calculated again (Supplementary Figure S3). In the future, the sources of the annotation data may accumulate rapidly. Therefore, we will continue to collect available resources, process these data to enrich the regulatory information and provide a new scoring strategy based on TF binding sites, eQTLs, enhancers and super enhancers, ATAC accessible regions and Hi-C considering tissue/cell-specific features. Moreover, we will add new methods of analysis in VARAdb such as upstream analysis. VARAdb will help users to predict the function of variation and provide perspectives of variation regulation.

## Supplementary Material

gkaa922_Supplemental_Files
