# Supplementary material for: VARAdb: a comprehensive variation annotation database for human
Source: Nucleic Acids Res. 2020 Oct 23;49(D1):D1431–44. doi: 10.1093/nar/gkaa922 (PMC7779011; doi:10.1093/nar/gkaa922)
Supplement: gkaa922_Supplemental_Files [file gkaa922_supplemental_files.zip › Supplementary Figure1.pdf]

Supplementary Figure 1. The case study of rs2279590 which is associated with Alzheimer's disease.

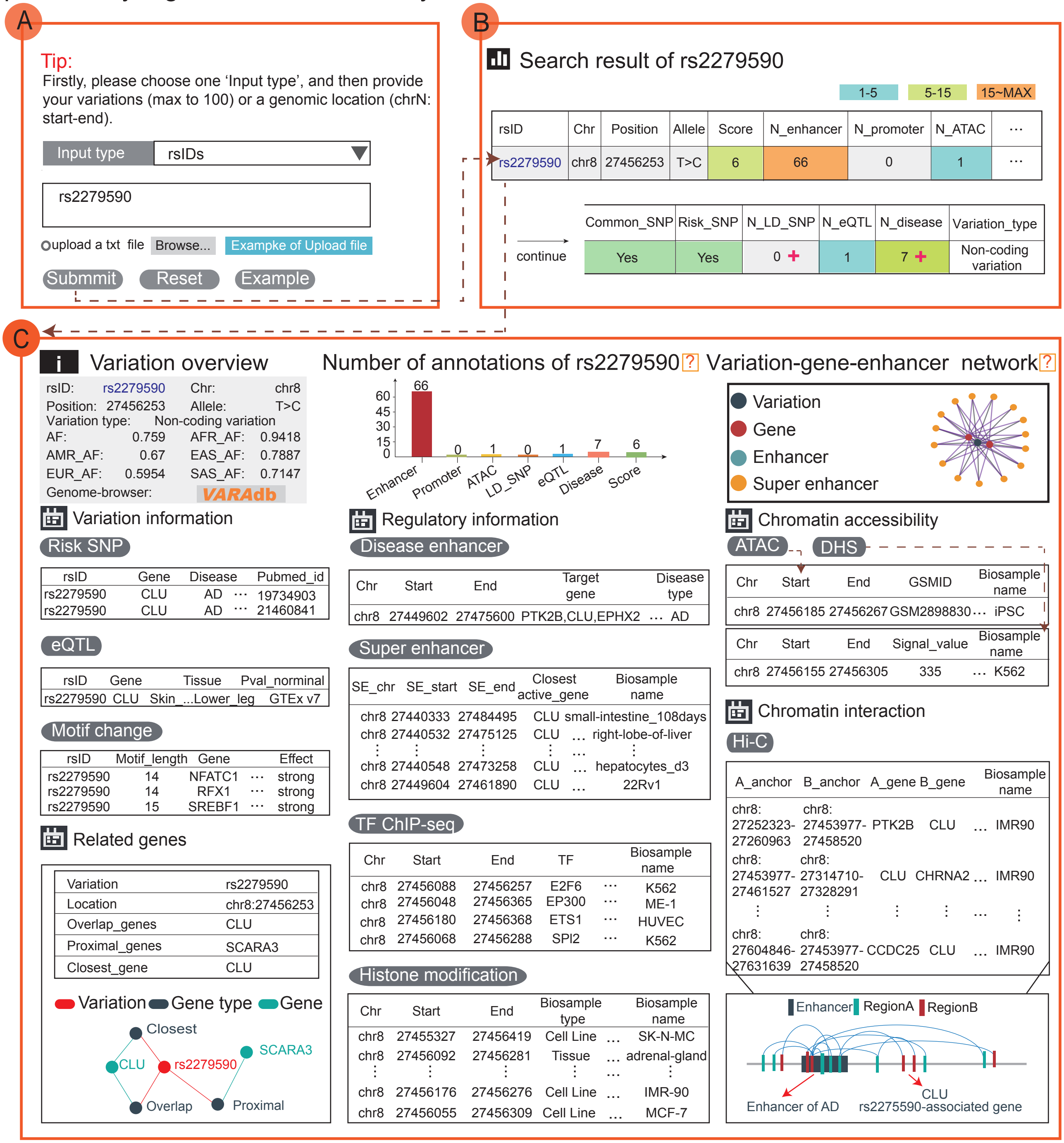

Supplementary Figure 1. The case study of rs2279590 which is associated with Alzheimer's disease (AD). (A) Input and parameters of 'Search by rsID or location'. (B) The summary table displays statistics about annotation information of rs2279590. (C) A detailed page of rs2279590, which provides an overview of rs2279590, variation-gene-enhancer network, and five annotation sections: 'Variation information', 'Related genes' (left panel), 'Regulatory information' (middle panel), 'Chromatin accessibility', and 'Chromatin interaction' (right panel).
