# Supplementary material for: VARAdb: a comprehensive variation annotation database for human
Source: Nucleic Acids Res. 2020 Oct 23;49(D1):D1431–44. doi: 10.1093/nar/gkaa922 (PMC7779011; doi:10.1093/nar/gkaa922)
Supplement: gkaa922_Supplemental_Files [file gkaa922_supplemental_files.zip › Supplementary Figure3.pdf]

Supplementary Figure 3. The workflow of updating the database.

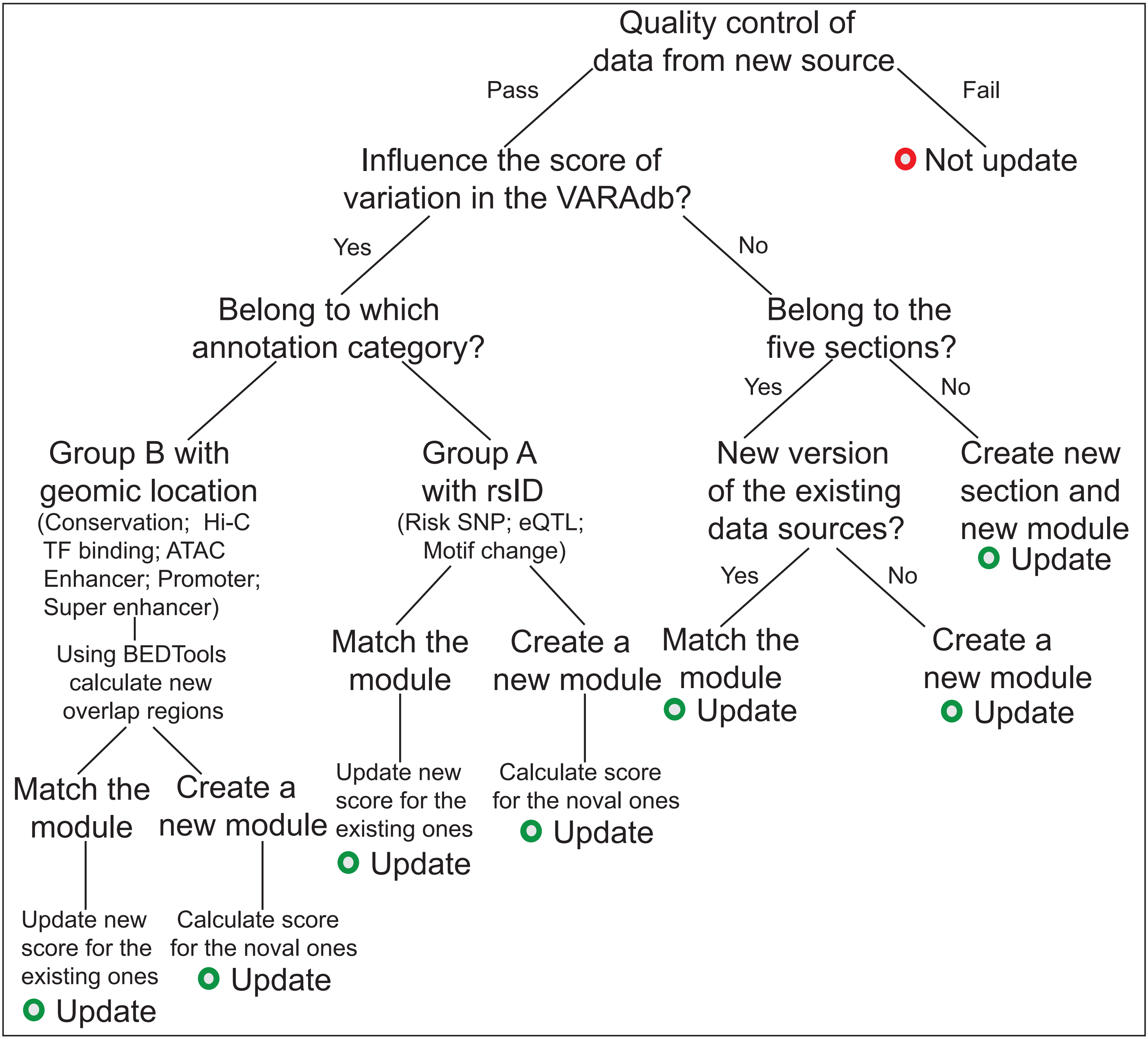

Supplementary Figure 3. The workflow of updating the database. When updating a data source, the quality of data will be first checked. If the passed data influence the score of variation in the database, the score will be calculated again. Otherwise, the data source will be added to the five sections or a new section. Then the data will be updated in a module way.
