# Supplementary material for: VARAdb: a comprehensive variation annotation database for human
Source: Nucleic Acids Res. 2020 Oct 23;49(D1):D1431–44. doi: 10.1093/nar/gkaa922 (PMC7779011; doi:10.1093/nar/gkaa922)
Supplement: gkaa922_Supplemental_Files [file gkaa922_supplemental_files.zip › Supplementary Material 1.docx]

**Supplementary Material 1 Description of the annotation number statistics of variation.**

To clarify the annotation number statistics for users, we descibe in details at the bottom of the summary table on the search results page as follows:

**1)rsID:** Reference SNP ID from dbSNP 151.

**2)Chr:** The chromosome of the variation.

**3)Position:** The position of the variation.

**4)Allele:** The ref allele > alt allele of the variation.

**5)Score:** The score of the variation.

**6)N_enhancer:** The number of enhancers and supers enhancers the variation locates in, including active enhancers from HACER GRO-seq/PRO-seq/FANTOM5 CAGE, disease enhancers from EnDisease and DiseaseEnhancer, super enhancers and typical enhancers from SEdb developed by our group previously, validated enhancers from VISTA Enhancer Browser and Endb, enhancer relevant states (“6_EnhG”; ”7_Enh”; “12_EnhBiv”) from Roadmap ChromHMM states.

**7)N_promoter:** The number of promoters the variation locates in, including promoter relevant states (“1_TssA ”; “2_TssAFlnk”) from Roadmap ChromHMM states and promoters from defined 2 kb upstream and 1 kb downstream of transcription start sites (TSSs) of GENCODE basic gene annotation file.

**8)N_ATAC:** The number of accessible chromatin regions the variation locates in from TCGA and Cistrome.

**9)Common SNP:** The variation is a common SNP from 1000 Genomes Project or not.

**10)Risk SNP:** The variation is a risk SNP from GWAS Catalog, GWASdbv2.0, GAD, a study of Johnson and O'Donnell and GRASP v2.0.

**11)N_LD_SNP:** LD SNPs of the variation. Click on the button to see specific LD SNPs.

**12)N_eQTL:** The number of eQTL pairs the variation owns from GTEx v7, PancanQTL and HaploReg v4.1.

**13)N_disease:** The diseases or traits or phenotypes are associated with the variation. These diseases/traits/phenotypes were integrated from DisGeNet 6.0, GWAS relevant information and cis-eQTL data of 33 cancers from TCGA.

**14) Variation_type:** Coding region variation or non-coding variation.
