# Supplementary figures and images for: VARAdb: a comprehensive variation annotation database for human
Source: Nucleic Acids Res. 2020 Oct 23;49(D1):D1431–44. doi: 10.1093/nar/gkaa922 (PMC7779011; doi:10.1093/nar/gkaa922)

Supplementary Figure 2. The case study of rs6983267 which is associated with colorectal cancer.

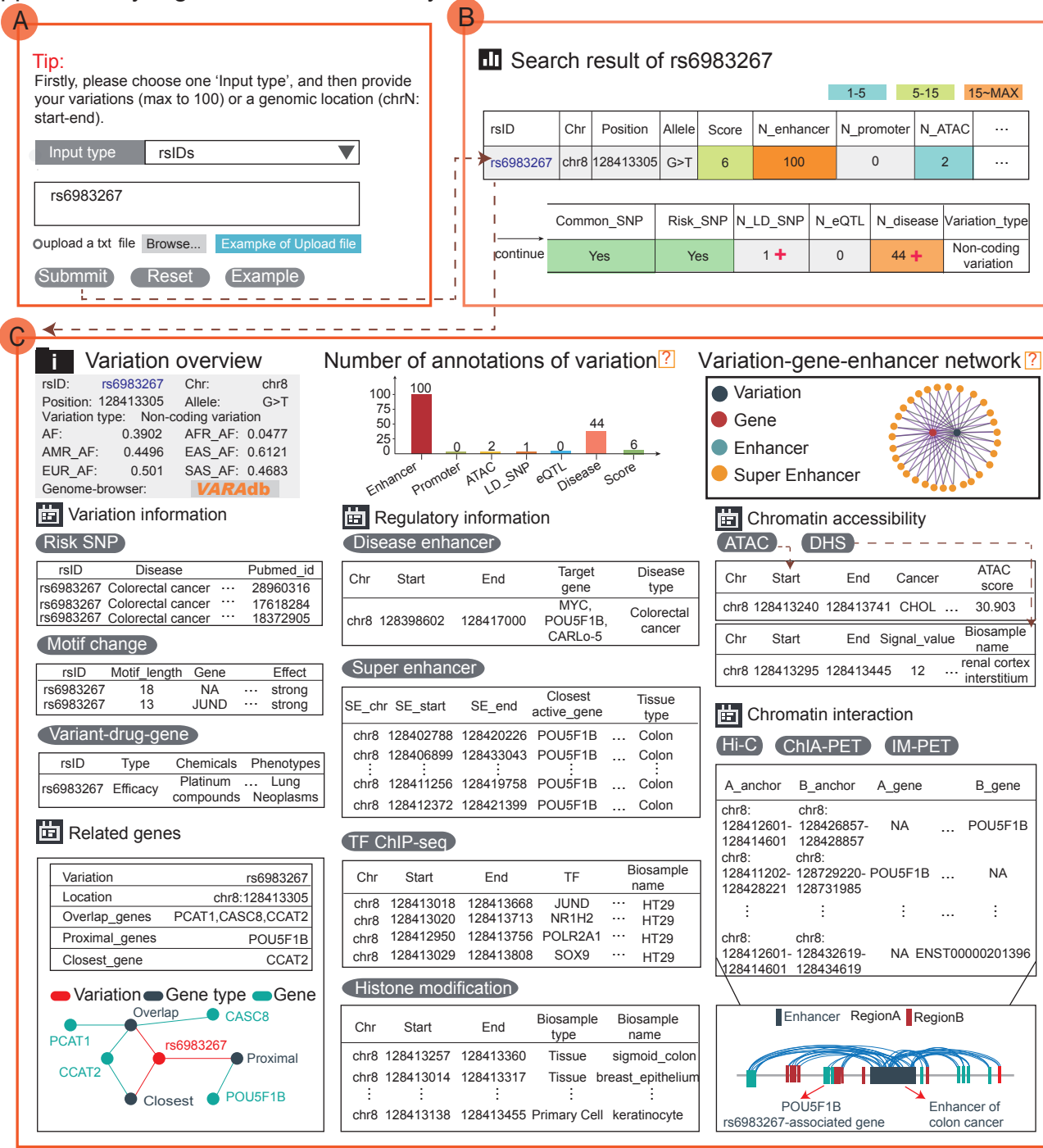

Supplement: gkaa922_Supplemental_Files [file gkaa922_supplemental_files.zip › Supplementary Figure2.pdf]
